# Simulated microgravity exposure markedly attenuates the baroreflex total loop gain and induces orthostatic hypotension in male rats

**DOI:** 10.14814/phy2.70637

**Published:** 2025-10-27

**Authors:** Kazuhiro Kamada, Hiroshi Mannoji, Takeshi Tohyama, Takuya Nishikawa, Hiroyuki Tsutsui, Kenji Sunagawa, Keita Saku

**Affiliations:** ^1^ Department of Cardiovascular Medicine Matsuyama Red Cross Hospital Matsuyama Ehime Japan; ^2^ Division of Cardiology Cardiovascular and Aortic Center Saiseikai Fukuoka General Hospital Fukuoka Japan; ^3^ Center for Clinical and Translational Research Kyushu University Hospital Fukuoka Japan; ^4^ Department of Research Promotion and Management National Cerebral and Cardiovascular Center Osaka Japan; ^5^ School of Medicine and Graduate School International University of Health and Welfare Fukuoka Japan; ^6^ Circulatory System Research Foundation Tokyo Japan; ^7^ Department of Cardiovascular Dynamics National Cerebral and Cardiovascular Center Osaka Japan

**Keywords:** arterial pressure, baroreflex, microgravity exposure, orthostatic hypotension, sympathetic nerve activity

## Abstract

Autonomic dysfunction is recognized as the central mechanism of orthostatic hypotension after microgravity exposure. Because the arterial baroreflex regulates arterial pressure (AP) via sympathetic nerve activity (SNA), we investigated the systematic baroreflex function under simulated microgravity by conducting an open‐loop analysis. We used Sprague–Dawley rats after hindlimb unloading (HU) for 2 weeks. We isolated the carotid sinuses from the systemic circulation, then changed intracarotid sinus pressure (CSP) stepwise and recorded SNA and AP responses. We compared CSP−AP (total loop), CSP−SNA (mechanoneural arc), and SNA−AP (neuromechanical arc) relations between control rats (*n* = 9) and HU rats (*n* = 7). The total loop gain at the operating point decreased significantly in HU rats compared with control rats (0.3 ± 0.4 vs. 1.1 ± 0.4 mmHg/mmHg, *p* < 0.05). In addition to the decrease in the mechanoneural arc gain at the operating point, the neuromechanical arc gain also decreased significantly in HU rats compared with control rats. A numerical simulation experiment showed a greater AP fall with a significant reduction of the total loop gain in response to hypotensive stress in HU rats. Thus, we conclude that simulated microgravity impairs baroreflex sympathetic regulation in the lower AP range, which may play an important role in the pathogenesis of orthostatic hypotension after microgravity exposure.

## 1 INTRODUCTION

Microgravity exposure induces orthostatic intolerance including dizziness and presyncope in 15%–60% of astronauts who returned to Earth (Yates & Kerman, 1998). It is well known that post‐spaceflight orthostatic hypotension (OH) is related to orthostatic intolerance, and a number of investigations have been performed to elucidate the physiological mechanisms of OH under actual or simulated microgravity. In addition to fluid shift toward the head, decreases in plasma volume, red blood cells, and total blood volume occur within days in spaceflight (Jordan et al., 2022). Circulating midregional pro‐atrial natriuretic peptide concentration decreases following a transient 80% increase on the first day in spaceflight (Drummer et al., 2001). Furthermore, actual or simulated microgravity enhances both resting and hypotension‐induced vasopressin levels (Mueller et al., 2006). Alterations in the regulation of the renin‐angiotensin system have been reported after spaceflight (Hughson et al., 2016). In addition to these hormonal changes, microgravity exposure can cause hemodynamic changes. Whether arterial pressure (AP) and heart rate (HR) change during spaceflight compared to pre‐flight levels remains controversial in humans (Eckberg et al., 2010; Norsk et al., 2015; Verheyden et al., 2010). On the other hand, the Bion‐M 1 space mission showed unchanged AP and increased HR in spaceflight mice during a 30‐day spaceflight and recovery compared with control mice (Andreev‐Andrievskiy et al., 2017). Microgravity exposure also affects autonomic functions. Ertl et al. (2002) reported that muscle sympathetic nerve activity (MSNA) and plasma noradrenaline concentration increased during spaceflight compared to pre‐flight, and that noradrenaline spillover remained elevated at 1–2 days post‐flight. Although the neck chamber technique is one of the methods to investigate carotid baroreceptor control of MSNA, AP, and HR (Rea & Eckberg, 1987), there were no studies to investigate MSNA response in microgravity, whereas Eckberg et al. (2010) demonstrated that in‐flight R‐R interval response to neck pressure changes declined from pre‐flight level. Thus, how microgravity exposure affects autonomic AP regulation via sympathetic nerve activity (SNA) remains largely unknown.

The arterial baroreflex function is a negative feedback system for stabilizing AP against disturbances, such as postural changes, via controlling SNA (Freeman, 2008). The lack of baroreflex function induces AP lability (Cowley Jr et al., 1973). Thus, several studies have investigated baroreflex sensitivity (BRS) in astronauts or simulated microgravity models to clarify the mechanism of OH. Fritsch et al. (1992) reported that short‐duration spaceflight (4–5 days) reduced post‐flight BRS relative to pre‐flight level. Cooke et al. (2000) demonstrated that long‐duration space mission (9 months) also reduced post‐flight BRS, with changes persisting as long as 2 weeks after returning to Earth. As simulated microgravity models, both head‐down‐tilt bed rest in humans and hindlimb unloading (HU) in rodents cause reduction of BRS (Hélissen et al., 2023; Kamiya et al., 2000; Moffitt et al., 1998). However, BRS does not accurately assess the baroreflex function of AP regulation, because it evaluates baroreflex under closed‐loop conditions.

To resolve the above limitation, Sato et al. (1999) proposed a novel framework to identify the baroreflex function of AP regulation by opening the negative feedback loop. This framework divides the arterial baroreflex system into two principal subsystems: a mechanoneural arc that describes how the baroreceptor pressure changes SNA, and a neuromechanical arc that describes how SNA changes AP.

We hypothesized that simulated microgravity exposure attenuates baroreflex function and increases AP lability. We investigated the impact of simulated microgravity exposure on the baroreflex‐mediated sympathetic AP regulation by performing a baroreflex open‐loop analysis in rats that had undergone HU for 2 weeks, which is considered to be a sufficient period to observe the effects of simulated microgravity on hemodynamics from the previous reports (Hélissen et al., 2023; Moffitt et al., 1998; Mueller et al., 2006). We also evaluated the AP variability in conscious, moving condition, which is an important index of arterial baroreflex function.

## 2 MATERIALS AND METHODS

### 2.1 Ethical approval

Animal care and experiments were approved by the Committee on Ethics of Animal Experiment, Kyushu University Graduate School of Medical Sciences, and were performed in accordance with the National Institutes of Health *Guide for the Care and Use of Laboratory Animals*. We used 8‐week‐old male Sprague–Dawley (SD) rats (Japan SLC, Hamamatsu, Japan). All rats were housed in a room maintained at a constant temperature (25 ± 2°C) with a 12:12‐h light–dark cycle. SD rats were fed a normal diet (5008 Formulab Diet, LabDiet, PMI Nutrition International). While HU rats were housed in individual cages, control rats were housed in groups without harnesses.

### 2.2 Hindlimb unloading

**FIGURE 1:**
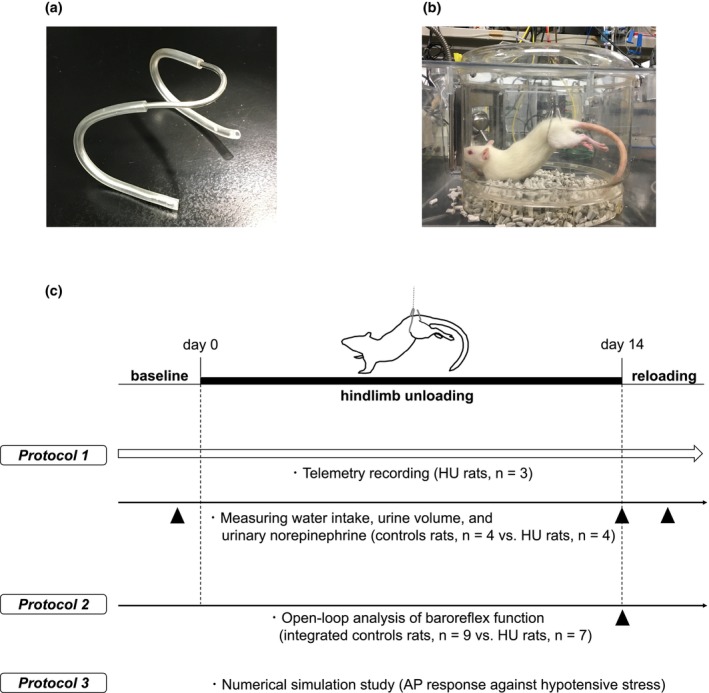
Hindlimb unloading and Protocols. (a) The pelvic support harness is made of stainless steel wire covered with an intravenous tube. (b) The harness is adjusted to the rat's body from the dorsum to the belly around both hind legs, and the rat is suspended at a head‐down tilt of approximately 30 degrees. (c) Schematic representation of the experimental design, Protocols 1–3. AP, arterial pressure; HU, hindlimb unloading.

We fabricated a pelvic support harness made of stainless steel wire (core diameter 2.0 mm) covered with an intravenous tube (Figure 1a). The harness was adjusted to the rat's body from the dorsum to the belly around both hind legs, and connected to a suspension mechanism. Rats were suspended at approximately a 30‐degree head‐down tilt in individual cages for 2 weeks (Figure 1b). After the HU period, the rats were returned to the normal four‐extremity weight‐bearing “reloaded” position for 1 day.

### 2.3 Urine volume, urinary norepinephrine, and water intake

We collected 24‐h urine samples using metabolic cages and measured urinary norepinephrine excretion. We also measured 24‐h water intake.

### 2.4 Echocardiography

Echocardiographic examinations (Aplio MX, Toshiba) were performed under a conscious state. We measured left ventricular (LV) wall diameters and LV dimensions.

### 2.5 Twenty‐four‐hour continuous AP recording

We implanted a telemetry system (TA11PA‐C40, Data Sciences, St. Paul, MN) in three rats. The catheter was inserted via the femoral artery and the catheter tip was placed in the abdominal aorta. The transmitter was implanted under the skin of the abdomen. We continuously recorded AP at a sampling frequency of 200 Hz.

### 2.6 Carotid sinus baroreceptor isolation

Rats were anesthetized by intraperitoneal injection (2 mL/kg) of a mixture of α‐chloralose (40 mg/mL) and urethane (250 mg/mL) and ventilated mechanically with oxygen‐enriched air. An appropriate level of anesthesia was maintained with intravenous infusion (2–3 mL•kg^−1^•h^−1^) of a 15‐fold diluted solution of the above anesthetic mixture during the experiment. Arterial pressure was measured using a high‐fidelity pressure transducer (SPR‐320, Millar Instruments, Houston, TX) inserted into the femoral artery. Body temperature was maintained at 37–38°C by a heating pad. A postganglionic branch of the splanchnic sympathetic nerve was exposed through a right flank incision. The splanchnic sympathetic nerve innervates the highly vascularized organs containing the majority of intravascular blood volume and responds similarly to the renal nerve against the central nervous system (Fudim et al., 2021; Kawada et al., 2010). A pair of stainless steel wire electrodes (Bioflex Wire AS633, Cooner Wire, Chatsworth, CA) was attached to the nerve to record SNA. The nerve and electrodes were secured and insulated with silicone glue (Kwik‐Sil, World Precision Instruments, Sarasota, FL). Pancuronium bromide (0.4 mg•kg^−1^•h^−1^) was infused continuously to prevent muscular activity from contaminating SNA. At the end of the experiment, a bolus injection of a ganglionic blocker hexamethonium bromide (60 mg/kg) was given to confirm the disappearance of SNA and to measure the background noise level of nerve signals.

To open the baroreflex feedback loop, we isolated the carotid sinus baroreceptors from the systemic circulation according to our previously reported methods (Saku et al., 2014). Briefly, a 7‐0 silk thread was passed between the external and internal carotid bifurcation. The internal carotid artery was embolized with stainless steel balls (0.8 mm in diameter, Tsubaki Nakashima, Nara, Japan). The isolated carotid sinuses were filled with physiological saline through catheters inserted into the common carotid arteries. Carotid sinus pressure (CSP) with a nonpulsatile signal was controlled using a high‐fidelity servo‐controlled piston pump (Model ET‐126A, Labworks, Costa Mesa, CA). Bilateral aortic depressor nerves and vagal nerves were sectioned at the neck to minimize reflex effects from the aortic arch and cardiopulmonary region.

### 2.7 Protocol

#### 2.7.1 Protocol 1: Analysis of SNA and continuous AP and HR in conscious, moving HU rats

We measured 24‐h urinary norepinephrine levels as an index of SNA at baseline (before HU), Day 14 of HU, and Day 1 after reloading in HU rats (*n* = 4) and compared them with control rats (*n* = 4) (Figure 1c). In addition, we recorded AP and HR for 24 h using the telemetry system, and compared mean values, SDs, and histograms derived from the telemetry data in HU rats (*n* = 3) at baseline; Day 1, Day 7, Day 14 of HU; and Day 1 after reloading.

#### 2.7.2 Protocol 2: Open‐loop analysis of baroreflex function

To estimate open‐loop static characteristics of the carotid sinus baroreflex, we measured the responses of SNA, AP, and HR to stepwise CSP increase, and compared the results between HU rats immediately after reloading following 14 days of HU (*n* = 7) and control rats without HU (control, *n* = 4; integrated control, *n* = 9) (Figure 1c). As mentioned in the statistics section below, we analyzed using the integrated control data (*n* = 9), in which the previous control data (*n* = 5) were added to the present control data (*n* = 4).

#### 2.7.3 Protocol 3: Numerical simulation study

**FIGURE 2:**
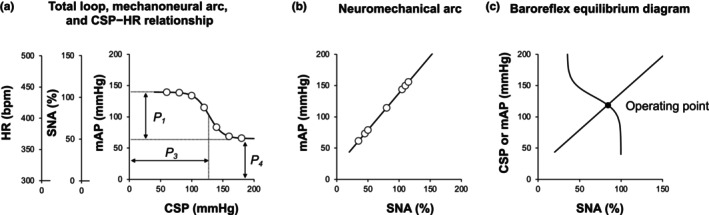
Analysis of open‐loop baroreflex function. (a) Baroreflex carotid sinus pressure (CSP)−arterial pressure (AP) relationship (total loop), CSP−sympathetic nerve activity (SNA) relationship (mechanoneural arc), and CSP−heart rate (HR) relationship each approximates an inverse sigmoid curve, and is quantified using a 4‐parameter logistic function (*P*_1_–*P*_4_). (b) SNA−AP relationship (neuromechanical arc) approximates a straight line and is quantified using linear regression. (c) The baroreflex equilibrium diagram is obtained by plotting the mechanoneural and neuromechanical arcs together on a pressure−SNA plane. The intersection between the mechanoneural and neuromechanical arcs is the operating point. *P*_1_, response range; *P*_2_, coefficient of slope; *P*_3_, midpoint of the operating range; *P*_4_, minimum value. mAP, mean arterial pressure.

To better understand AP regulation after simulated microgravity exposure, we performed a numerical simulation of the responses in baroreflex function and AP under hypotensive stress (−30 mmHg) based on the open‐loop characteristics of the baroreflex obtained in protocol 2. Sato et al. (1999) reported that withdrawal of circulating blood volume shifted the neuromechanical arc downward without changing the gain of the mechanoneural arc. Therefore, we calculated the total loop gain of baroreflex and operating point by shifting the neuromechanical arc downward (−30 mmHg) in each of the rats in protocol 2, integrated control rats (*n* = 9) and HU rats (*n* = 7) (Figure 2).

### 2.8 Data analysis

In protocol 1, we recorded AP and HR at 200 Hz. We used continuous 24‐h recordings of AP and HR for analysis. After downsampling the data at 1 Hz, we generated histograms of mean AP and HR at intervals of 1 mmHg and 1 beat/min, respectively.

In protocol 2, experimental data were recorded at 200 Hz using a 16‐bit analog‐to‐digital converter (Power Laboratory 16/35. AD Instruments, Sydney, NSW, Australia) and stored in a dedicated laboratory computer system. To quantify SNA, preamplified nerve signals were bandpass filtered at 150–1000 Hz, and then full‐wave rectified and low‐pass filtered at a cutoff frequency of 30 Hz using analog circuits. We increased CSP stepwise from 60 to 180 mmHg every 60 s which was sufficient for the hemodynamic responses to stabilize. Mean SNA, AP, and HR were calculated at each CSP level by averaging the data during the last 10 s of each step. Because the absolute amplitude of SNA varied among animals depending on the recording conditions, SNA was standardized in each animal as follows: SNA corresponding to the CSP level of 60 mmHg was assigned a value of 100%, and the noise level after hexamethonium administration was assigned a value of 0%. The data obtained from three consecutive stepwise CSP inputs were averaged for analysis.

The CSP–AP relationship (baroreflex total loop), CSP–SNA relationship (baroreflex mechanoneural arc), and CSP–HR relationship each approximated an inverse sigmoid curve (Figure 2a) and were quantified using a four‐parameter logistic function as follows (Kawada & Sugimachi, 2016):

$$y=\frac{P_{1}}{1+\exp\left[P_{2}\left(x-P_{3}\right)\right]}+P_{4}$$

where *x* and *y* represent the input (CSP) and output (SNA, mean AP, or HR) values, respectively, *P*_*1*_ is the response range of *y* (the difference between the maximum and minimum values of *y*), *P*_*2*_ is the slope coefficient, *P*_*3*_ is the midpoint of the sigmoid curve on the CSP axis, and *P*_*4*_ is the minimum value of *y* (the lower plateau of the sigmoid curve). The maximum gain or maximum slope of the sigmoid curve (*G*_max_) was calculated as follows:

$$G_{\max}=P1\times P2/4$$

**FIGURE 3:**
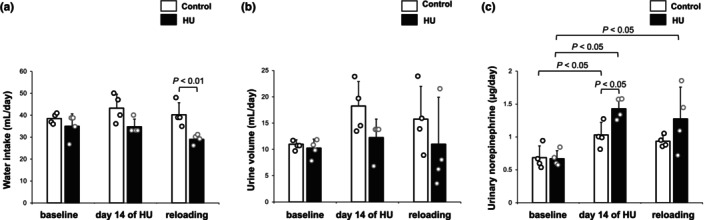
Effects of hindlimb unloading (HU) on water intake, urine volume, and 24‐h urinary norepinephrine. (a) Water intake, (b) urine volume, and (c) 24‐h urinary norepinephrine are compared between control rats and HU rats at baseline, Day 14 of HU, and Day 1 after reloading. Data are expressed as mean ± SD (control, *n* = 4; HU, *n* = 4). Data of each rat are shown as each dot.

For the total loop, the operating point was calculated from the intersection between the sigmoid curve and the line of identity (CSP = mean AP). The total loop gain of baroreflex at the operating point was calculated as the slope of the sigmoid curve (ΔAP/ΔCSP) at the operating point. Furthermore, the total loop gains of baroreflex in the lower AP range (80–120 mmHg) were calculated by averaging the gains obtained at CSP of 80–120 mmHg.

The SNA–AP relationship (baroreflex neuromechanical arc) approximated a straight line (Figure 2b) and was quantified using linear regression as follows (Kawada & Sugimachi, 2016):

meanAP=a×SNA+b

where *a* and *b* are the slope and intercept, respectively.

The baroreflex equilibrium diagram is obtained by plotting the mechanoneural and neuromechanical arcs together on a pressure–SNA plane (Figure 2c). The intersection between the mechanoneural and neuromechanical arcs is the operating point, at which SNA and AP are regulated around these values when the baroreflex negative feedback loop is closed. The total loop gain of the baroreflex at the operating point is calculated from the product of the tangential slope of the mechanoneural arc (ΔSNA/ΔCSP) and the slope of the neuromechanical arc (ΔAP/ΔSNA).

### 2.9 Statistics

All data are presented as mean ± standard deviation (SD) in each analysis. Differences between control rats and HU rats were assessed using an unpaired Student's *t*‐test. Differences among the same group in protocol 1 (water intake, urine volume, and urinary norepinephrine) and protocol 2 (mean AP, HR, and the respective distribution) were evaluated by one‐way analysis of variance (ANOVA), and Tukey–Kramer test was used for post hoc comparisons. JMP software (version 17, SAS Institute) was used in statistical analyses. Differences were considered significant when *p* < 0.05. Based on the results of similar previous experiments, which showed minimal variability in the control group, we initially conducted statistical analysis with a control group (*n* = 4). However, when comparing the control group with the HU group, the statistical differences and equivalence were unclear due to insufficient power. Consequently, we incorporated data from our prior control group data (*n* = 5) (Saku et al., 2017), increasing the control group size to *n* = 9 for the final analysis.

## 3 RESULTS

### 3.1 Baseline characteristics and 24‐h urinary norepinephrine

As shown in Table 1, body weight and soleus muscle weight in HU rats (Day 14 of HU) were significantly lower compared with control rats. When expressed relative to body weight, the soleus‐to‐body mass ratio was reduced by 39% following HU. This result indicates that HU leads to significant atrophy in the soleus muscle. There were no significant differences in echocardiographic data between control and HU rats. Water intake in HU rats on day 14 of HU tended to be lower compared with control rats (34.8 ± 3.5 vs. 43.3 ± 6.4 mL/day, *p* = 0.06), and that on Day 1 after reloading was significantly lower compared with control rats (Figure 3a). Urine volume in HU rats on Day 14 of HU tended to be lower compared with control rats (12.3 ± 3.5 vs. 18.3 ± 4.6 mL/day, *p* = 0.08), but the difference was not significant (Figure 3b). Although 24‐h urinary norepinephrine excretion increased with time after baseline measurement both in control rats and HU rats, HU rats on Day 14 of HU showed significantly higher 24‐h urinary norepinephrine excretion compared with control rats (1.4 ± 0.2 vs. 1.0 ± 0.2 μg/day, *p* < 0.05). On Day 1 after reloading, 24‐h urinary norepinephrine excretion was not significantly different between HU and control groups, while the level in HU rats remained significantly increased compared with baseline (Figure 3c).

**TABLE 1:** Animal characteristics and echocardiography in control rats and rats on day 14 of hind‐limb unloading.

	Control rats	HU rats (day 14 of HU)
Number of rats	4	6–7
Body weight, g	341 ± 11	301 ± 7*
Soleus muscle weight, mg	241 ± 13	131 ± 10*
Soleus‐to‐body mass ratio, mg/kg	707 ± 58	433 ± 32*
Echocardiography		
Anterior wall diameter, mm	1.9 ± 0.2	1.6 ± 0.3
Posterior wall diameter, mm	1.8 ± 0.3	2.1 ± 0.4
Left ventricular end‐diastolic dimension, mm	6.6 ± 0.4	6.7 ± 0.6
Left ventricular end‐systolic dimension, mm	2.3 ± 0.5	2.6 ± 0.7
Left ventricular fractional shortening, %	64.8 ± 5.9	61.9 ± 8.8

*Note*: Data are presented as means ± SD.

Abbreviation: HU, hind‐limb unloading.

* *p* < 0.0001, significantly different from control rats, unpaired Student's *t*‐test.

### 3.2 Effects of simulated microgravity on AP and HR variabilities in conscious moving HU rats

**FIGURE 4:**
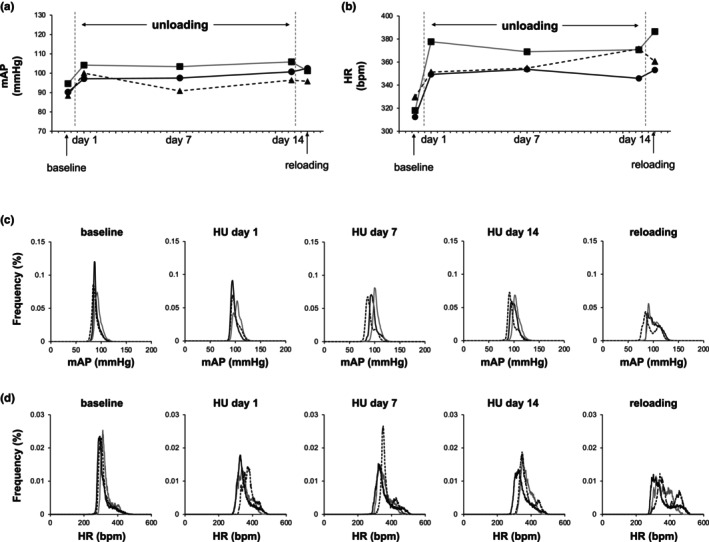
Hemodynamic changes during hindlimb unloading (HU) and after reloading. Changes in (a) mean arterial pressure (mAP) and (b) heart rate (HR) at baseline, Day 1 of HU, Day 7 of HU, Day 14 of HU, and Day 1 after reloading. Data were obtained from 3 HU rats and are expressed as solid circle (●), solid triangle (▲), and solid square (■), respectively. Changes in (c) mAP histograms and (d) HR histograms of individual rats at baseline, Day 1 of HU, Day 7 of HU, Day 14 of HU, and Day 1 after reloading. Data of each rat are shown (rat 1: black solid line, rat 2: gray solid line, rat 3: dotted line).

Figure 4a,b show mean AP and HR in HU rats at baseline; Day 1, Day 7, and Day 14 of HU; and Day 1 after reloading. The mean AP at Day 1 and Day 14 of HU tended to be higher compared with baseline, but the differences were not significant (*p* = 0.08 and 0.06, respectively) (Figure 4a and Table 2). On the other hand, mean HR was significantly higher compared with baseline during the entire HU period and after reloading (Figure 4b and Table 2). Histograms of mean AP and HR from individual HU rats are shown in Figure 4c,d, respectively. Hindlimb unloading significantly enhanced AP variability compared to baseline, and the distribution of mean AP was dispersed markedly after reloading (Figure 4c and Table 2). The distribution of HR also showed significant dispersion after reloading compared with baseline (Figure 4d and Table 2).

**TABLE 2:** Mean values and standard deviation (SD) of arterial pressure and heart rate obtained from continuous recording in hind‐limb unloading rats.

	HU rats (*n* = 3)				
	Baseline	Unloading			Reloading
		Day 1	Day 7	Day 14	
AP mean, mmHg	91 ± 3.2	100 ± 4	97 ± 6	101 ± 5	100 ± 4
SD, mmHg	6.7 ± 0.5	7.2 ± 1.0^†^	7.6 ± 0.9^†^	7.6 ± 0.5^†^	12.0 ± 1.8^‡^
HR mean, bpm	320.1 ± 8.8	359.4 ± 15.8*	359.1 ± 8.5*	362.7 ± 14.5*	366.8 ± 17.5*
SD, bpm	34.1 ± 2.3	36.3 ± 3.6	38.7 ± 7.4	37.6 ± 7.0	51.8 ± 9.7*

*Note*: Data are presented as means ± SD. *p < 0.05, ^†^ *p* < 0.01, ^‡^ *p* < 0.001, significantly different from baseline, one‐way factorial analysis of variance followed by post hoc Tukey–Kramer tests.

Abbreviations: AP, arterial pressure; HR, heart rate; HU, hind‐limb unloading.

### 3.3 Effects of simulated microgravity on baroreflex static characteristics

**FIGURE 5:**
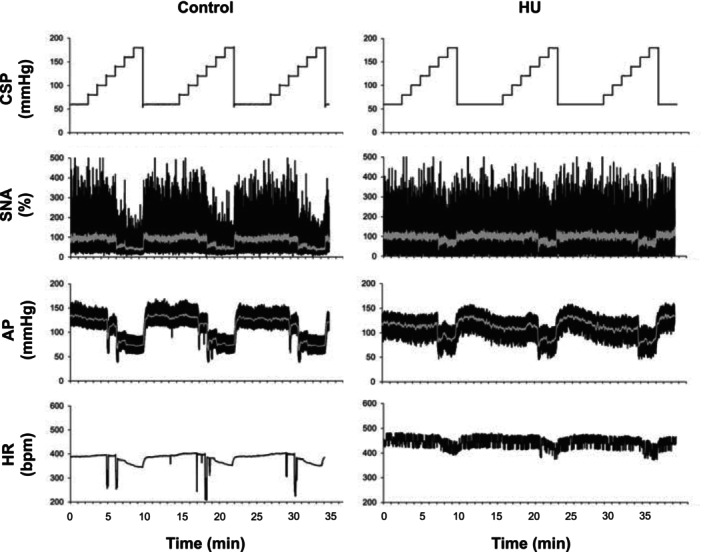
Representative recordings of hemodynamics under baroreflex open‐loop conditions in a control rat and a hindlimb unloading (HU) rat. Carotid sinus pressure (CSP) was decoupled from arterial pressure (AP) and changed stepwise from 60 to 180 mmHg to characterize the baroreflex function. Gray lines in sympathetic nerve activity (SNA) and AP recordings indicate 1‐s moving averaged data. HR, heart rate.

Figure 5 shows the representative time series of SNA and hemodynamics in a control rat and a HU rat. In the control rat, the stepwise increase in CSP decreased SNA, AP, and HR. In the HU rat, however, SNA, AP, and HR did not change during the initial stepwise increase of CSP from 60 to140 mmHg, but all three variables decreased with further CSP increase up to 180 mmHg.

**FIGURE 6:**
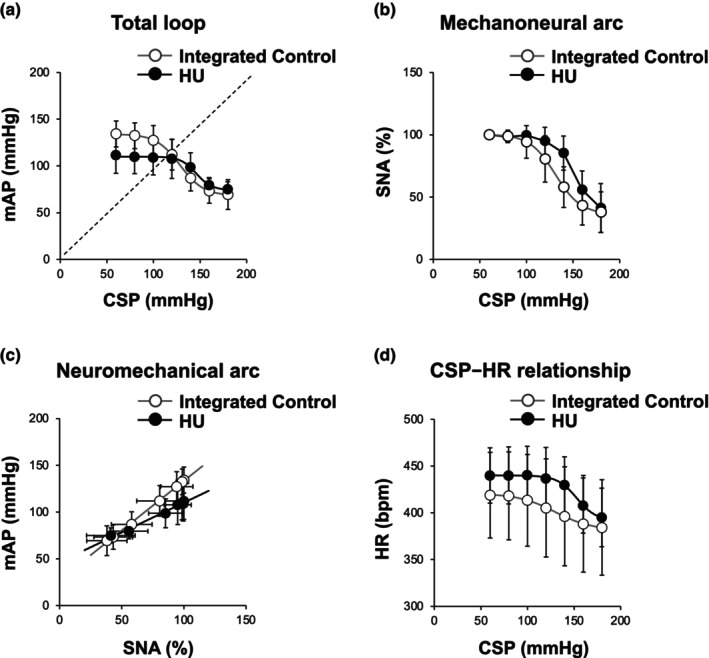
Open‐loop static characteristics of the baroreflex obtained from integrated control rats and hindlimb unloading (HU) rats. (a) Baroreflex total loop carotid sinus pressure (CSP)−arterial pressure (AP) relationship, (b) baroreflex mechanoneural arc (CSP−SNA relationship), (c) baroreflex neuromechanical arc (SNA−AP relationship), and (d) CSP−heart rate (HR) relationship. Data are expressed as mean ± SD (integrated control, *n* = 9; HU, *n* = 7). mAP, mean arterial pressure.

Figure 6 shows the group‐averaged open‐loop static characteristics of the baroreflex obtained from integrated control rats and HU rats, and Table 3 shows the estimated parameters of open‐loop static characteristics. In both groups, the baroreflex total loop approximated an inverse sigmoid curve (Figure 6a). For the total loop, there were no significant differences in the response range of AP (*P*_*1*_), the slope coefficient (*P*_*2*_), and the minimum value of AP (*P*_*4*_) between the HU rats and control rats. The sigmoid curve on the CSP axis (*P*_*3*_) was significantly higher in HU rats than in control rats. In comparison with integrated control rats, HU rats showed a significant decrease in *P*_*1*_, adding the same trend in *P*_*2*_, *P*_*3*_, and *P*_*4*_ as shown in the comparison with control rats. Consequently, there was no significant difference in the maximum gain of the sigmoid curve (*G*_max_) between control or integrated control rats and HU rats, whereas the total loop gain at the operating point decreased significantly in HU rats compared with control or integrated control rats. The average total loop gain in the lower AP range (80–120 mmHg) was markedly reduced in HU rats compared with integrated control rats (0.12 ± 0.14 vs. 0.64 ± 0.14 mmHg/mmHg, *p* < 0.001).

**TABLE 3:** Parameters of open‐loop static characteristics of the baroreflex in control rats, integrated control rats, and hindlimb unloading rats.

	Control	Integrated control	HU
Number of rats	4	9	7
Total loop			
*P*_1_, mmHg	53.2 ± 8.3	70.4 ± 26.3	37.4 ± 17.1^†^
*P*_2_, mmHg/mmHg	0.13 ± 0.01	0.09 ± 0.04	0.18 ± 0.18
*P*_3_, mmHg	129.3 ± 2.7	128.5 ± 7.2	144.2 ± 9.5*^,‡^
*P*_4_, mmHg	81.9 ± 11.1	65.4 ± 20.7	73.9 ± 8.7
*G*_max_, mmHg/mmHg	1.6 ± 0.2	1.5 ± 0.3	1.5 ± 1.1
Operating point			
AP, mmHg	119.7 ± 7.3	115.7 ± 7.7	108.5 ± 16.1
Total loop gain, mmHg/mmHg	1.1 ± 0.7	1.1 ± 0.4	0.3 ± 0.4*^,‡^
Mechanoneural arc			
*P*_1_, %	52.6 ± 15.9	67.3 ± 18.3	62.4 ± 21.1
*P*_2_, %/mmHg	0.13 ± 0.05	0.09 ± 0.05	0.18 ± 0.19
*P*_3_, mmHg	134.1 ± 2.6	129.7 ± 12.2	150.7 ± 6.4**^,‡^
*P*_4_, %	49.9 ± 16.3	35.5 ± 17.9	38.0 ± 21.1
*G*_max_, %/mmHg	1.7 ± 0.7	1.4 ± 0.5	2.2 ± 1.3
Neuromechanical arc			
*a*, mmHg/%	0.9 ± 0.3	1.0 ± 0.2	0.6 ± 0.2*^,‡^
*b*, mmHg	35.1 ± 29.8	32.5 ± 22.4	46.3 ± 16.4
CSP−HR relationship			
*P*_1_, beats/min	40.7 ± 19.7	45.9 ± 25.6	58.7 ± 16.1
*P*_2_, beats•min^−1^•mmHg^−1^	0.11 ± 0.01	0.08 ± 0.04	0.09 ± 0.05
*P*_3_, mmHg	140.2 ± 3.7	123.2 ± 18.0	160.3 ± 20.1^‡^
*P*_4_, beats/min	409.1 ± 58.3	376.3 ± 49.9	384.1 ± 29.5
*G*_max_, beats•min^−1^•mmHg^−1^	1.1 ± 0.5	0.8 ± 0.4	1.3 ± 0.9
Baroreflex equilibrium diagram			
Operating point AP, mmHg	115.6 ± 5.0	117.3 ± 9.8	107.7 ± 16.8
Operating point SNA, %	89.5 ± 12.8	81.3 ± 12.1	98.8 ± 4.8^‡^
Operating point Total loop gain, mmHg/mmHg	1.2 ± 0.7	1.1 ± 0.5	0.3 ± 0.4*^,‡^

*Note*: Data are presented as means ± SD. HU, hindlimb unloading; *P*_1_–*P*_4_, parameters of a four‐parameter logistic function of inverse sigmoid curve (*P*_1_, response range; *P*_2_, coefficient of slope; *P*_3_, midpoint of the operating range; *P*_4_, minimum value); *G*_max_, maximum gain calculated from *P*_1_ × *P*_2_/4. a and b are parameters calculated from linear regression (a, slopes; b, intercepts). Plots of CSP−AP relationship (total loop), CSP−SNA relationship (mechanoneural arc), SNA−AP relationship (neuromechanical arc), and CSP−HR relationships are plotted in Figure 6. In the baroreflex total loop, the gain at the operating point was calculated from the slope (ΔAP/ΔCSP) at the intersection of the fitted curve of the total loop and the identity line (AP = CSP). In the baroreflex equilibrium diagram (see Figure 7), total loop gain at the operating point was calculated from the product of the slope of the mechanoneural arc (ΔSNA/ΔCSP) and the slope of the neuromechanical arc (ΔAP/ΔSNA). *p < 0.05, **p < 0.01, significantly different from control rats, unpaired Student's *t*‐test. ^†^ *p* < 0.05, ^‡^ *p* < 0.01 significantly different from integrated control rats, unpaired Student's *t*‐test.

Abbreviations: AP, arterial pressure; CSP, carotid sinus pressure; SNA, sympathetic nerve activity.

The baroreflex mechanoneural arc also showed an inverse sigmoidal curve in both groups (Figure 6b). For the mechanoneural arc, there were no significant differences in *P*_*1*_, *P*_*2*_, and *P*_*4*_, but *P*_*3*_ was significantly higher in HU rats than in control or integrated control rats (Table 3). In the neuromechanical arc, the slope (*a*) was significantly lower in HU rats than in control or integrated control rats, whereas there was no significant difference in the intercept (*b*) between control or integrated control rats and HU rats (Table 3).

The static CSP–HR relationship showed an inverse sigmoidal curve (Figure 6d). Although the midpoint of the sigmoid curve on the CSP axis (*P*_*3*_) tended to be higher in HU rats than in control rats, this showed a significant difference in HU rats compared with integrated control rats. There were no significant differences in the other parameters (*P*_*1*_, *P*_*2*_, and *P*_*4*_) between control or integrated control rats and HU rats (Table 3).

**FIGURE 7:**
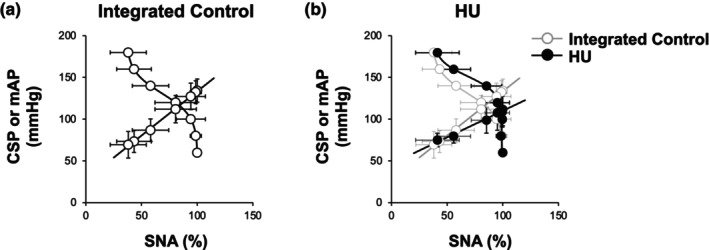
Averaged baroreflex equilibrium diagrams obtained from integrated control rats and hindlimb unloading (HU) rats. Averaged baroreflex equilibrium diagram obtained from (a) integrated control rats and (b) HU rats. Gray circles and lines show the data of integrated control rats. Data are expressed as mean ± SD (integrated control, *n* = 9; HU, *n* = 7). CSP, carotid sinus pressure; mAP, mean arterial pressure; SNA, sympathetic nerve activity.

Figure 7a,b show the baroreflex equilibrium diagrams. Although there was no significant difference in operating point AP, operating point SNA tended to be higher in HU rats than in control rats. The difference in operating point SNA became remarkable when compared with integrated control rats (Table 3). The mechanoneural arc shifted upward (toward higher CSP) and the slope of the neuromechanical arc decreased in HU rats. As a result, the total loop gain at the operating point decreased significantly in HU rats compared with control or integrated control rats.

### 3.4 Numerical simulation study

**FIGURE 8:**
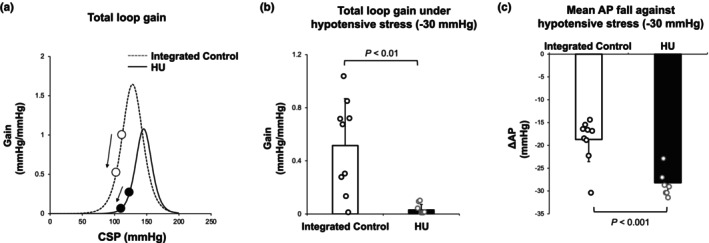
Numerical simulation of baroreflex responses against hypotensive stress in integrated control rats and hindlimb unloading (HU) rats. (a) Changes in total loop gain of baroreflex under simulated hypotensive stress of −30 mmHg in integrated control rats and HU rats. The total loop gain of baroreflex at the operating point decreases as shown by the arrow in each graph. (b) Calculated total loop gain of baroreflex under hypotensive stress (−30 mmHg) in integrated control rats (*n* = 9) and HU rats (*n* = 7). (c) Estimated mean arterial pressure fall (ΔAP) against hypotensive stress (−30 mmHg) in integrated control rats (*n* = 9) and HU rats (*n* = 7). Data are expressed as mean ± SD. Data of each rat are shown as each dot. AP, arterial pressure; CSP, carotid sinus pressure.

In the numerical simulation study using the baroreflex data obtained in protocol 3, we shifted the neuromechanical arc downward (−30 mmHg) to mimic hypotensive stress. As shown in Figure 8a, the hypotensive stress decreased the total loop gain in both groups. The hypotensive stress (−30 mmHg) caused a significant decrease in total loop gain in HU rats compared with integrated control rats (HU vs. integrated control: 0.03 ± 0.04 vs. 0.52 ± 0.35 mmHg/mmHg, *p* < 0.01) (Figure 8b). The change in the operating point AP against hypotensive stress (−30 mmHg) was significantly greater in HU rats than in integrated control rats (HU vs. integrated control: −28.1 ± 2.7 vs. −18.7 ± 4.9 mmHg, *p* < 0.001) (Figure 8c).

## 4 DISCUSSION

To the best of our knowledge, this is the first comprehensive study elucidating open‐loop characteristics of the arterial baroreflex in HU rats as a model of simulated microgravity. The major findings are as follows. First, the total loop gain of the baroreflex at the operating point decreased significantly in HU rats compared with control rats. This characteristic could explain the finding that AP distribution was dispersed markedly in HU rats after reloading compared with baseline under conscious, moving conditions. Furthermore, the total loop gain of the baroreflex in the lower AP range was significantly reduced in HU rats compared with control rats. Second, the baroreflex mechanoneural arc shifted toward higher CSP in HU rats than in control rats. In addition, the slope of the baroreflex neuromechanical arc decreased significantly in HU rats compared with control rats. Changes in both the baroreflex mechanoneural and neuromechanical arcs in HU rats led to decreases in the respective slopes at the operating point, resulting in the reduction of the total loop gain of the baroreflex at the operating point. Finally, a numerical simulation study corroborated that hypotensive stress induced a significant reduction in the total loop gain of the baroreflex and a decrease in the operating point AP in HU rats compared with control rats.

### 4.1 Effects of simulated microgravity on hemodynamics

The present study is the first to provide physiological evidence for impaired blood pressure regulation after simulated microgravity exposure through conducting an open‐loop analysis of baroreflex. Hindlimb unloading has been used to investigate the effects of microgravity in rodents. This model induces a cephalic fluid shift to the upper part of the body and causes muscle and bone atrophy (Chowdhury et al., 2013; Martel et al., 1996). There are several similarities between HU and spaceflight models, including responses in heart, pulmonary, intestine, immune, and endocrine functions (Morey‐Holton & Globus, 2002). To investigate the effects of HU on hemodynamics, we recorded AP and HR in conscious, moving HU rats in protocol 1. Mean AP tended to be elevated from baseline during HU and after reloading, although the differences did not reach statistical significance (Figure 4a and Table 2). Furthermore, HR increased significantly from baseline during HU and after reloading (Figure 4b and Table 2). The present results were similar to previous studies using HU rats (Hélissen et al., 2023; Moffitt et al., 2008; Tsvirkun et al., 2012). Furthermore, the Bion‐M 1 space mission using mice also showed data consistent with the present results. In addition to the unchanged AP and increased HR during spaceflight, the study showed a steeper HR increase in spaceflight mice during running after spaceflight compared with control mice (Andreev‐Andrievskiy et al., 2017). These results indicate that microgravity exposure can augment sympathetic, and diminish vagal cardiovascular influences. As will be explained later, actual or simulated microgravity can cause cardiac atrophy and vascular remodeling (Bederman et al., 2015; Jung et al., 2005; Perhonen et al., 2001; Summers et al., 2005). Therefore, we speculate that it may be necessary to activate SNA and increase HR to maintain cardiac output. Regarding the activated SNA, hindlimb unloading caused a significant increase in 24‐h urinary norepinephrine excretion that reflects SNA, compared with control rats in the present study (Figure 3c). This result is consistent with the previous study which reported that plasma norepinephrine and epinephrine concentrations of astronauts increased 34% and 65%, respectively, on the day of landing after 4.9–13.8 days of Space Shuttle missions compared with before flight (Whitson et al., 1995).

### 4.2 Open‐loop characteristics of the arterial baroreflex in HU rats

The arterial baroreflex stabilizes AP against disturbances such as postural changes. Because microgravity exposure is well known to induce orthostatic intolerance, we hypothesized that the arterial baroreflex function, that is, the total loop gain of baroreflex, would be attenuated in HU rats. In protocol 1, HU rats showed markedly dispersed AP distribution after reloading, implying that HU attenuated the baroreflex function. In protocol 2, there was no significant difference in *G*_max_ between HU rats and integrated control rats, but the total loop gain of baroreflex at the operating point decreased significantly in HU rats compared with integrated control rats. Furthermore, the midpoint of the sigmoid curve of the total loop gain of baroreflex was shifted toward a higher CSP range. This alteration resulted in attenuation of the total loop gain of baroreflex in the lower AP range in HU rats compared with integrated control rats.

We divided the baroreflex system into two subsystems; that is, mechanoneural and neuromechanical arcs, to understand the total loop function, because the product between the slope of the mechanoneural arc (ΔSNA/ΔCSP) and the slope of the neuromechanical arc (ΔAP/ΔSNA) gives the total loop gain of baroreflex. Since the neuromechanical arc approximated linear characteristics, the sigmoidal characteristics of the baroreflex total loop were derived from the mechanoneural arc. In HU rats, the midpoint of the sigmoid curve of the mechanoneural arc was shifted significantly upward compared with integrated control rats, and the sigmoid curve was shifted toward higher CSP range, similar to the findings in the baroreflex total loop. The neuromechanical arc describes the AP response to SNA. As expected, the neuromechanical arc in HU rats was shifted downward in the present study. The total loop gain at the operating point significantly reduced compared with the *G*_max_ both in integrated control rats and HU rats, because the operating point deviates from the midpoint of the mechanoneural arc. However, the degree of reduction of the gain at the operating point from the *G*_max_ was larger in HU rats than in integrated control rats. We consider that the mechanoneural and neuromechanical arcs abnormally intersected in HU rats in addition to the changes in the properties of each arc.

There are several possible mechanisms for the alterations in the mechanoneural arc. Yamasaki et al. (2004) reported that rats housed for 16 days under microgravity in space showed a decrease in the number of unmyelinated fibers in the aortic depressor nerve. Although this histological change has not been demonstrated in HU rats, this mechanism could have contributed to the change in the mechanoneural arc observed in this study. Additionally, the change in the rostral ventrolateral medulla (RVLM), a primary site in the central nervous system for SNA control, has been reported in HU rats. Moffitt et al. (2002) demonstrated that γ‐aminobutyric acid receptor‐mediated inhibition of the RVLM was enhanced after HU. The augmented inhibition was not dependent on input from the caudal ventrolateral medulla. Although the nucleus tractus solitarius (NTS) is the first central site where autonomic and cardiovascular reflex functions are integrated, Lima‐Silveira et al. (2020) have shown that HU induces synaptic and neuronal plasticity in NTS neurons mono‐ and poly‐synaptically connected with tractus solitarius. Further investigations may be needed to clarify the mechanisms of changes in the mechanoneural arc in HU rats.

The neuromechanical arc is involved in cardiac function, vascular properties, and stressed volume. Some studies in humans subjected to microgravity or head‐down‐tilt bed rest demonstrated cardiac atrophy (Perhonen et al., 2001; Summers et al., 2005). Hindlimb unloaded rodent models also showed significant cardiac atrophy after HU (Bederman et al., 2015). In the present study, there was no significant difference in LV fractional shortening between HU and control rats. However, measurement of end‐systolic elastance (*E*_es_) is needed to accurately assess myocardial contractility. Jung et al. (2005) constructed pressure–volume loops in HU mice, and found significant decreases in both *E*_es_ and effective arterial elastance (*E*_a_) in HU mice compared with denervated controls. Additionally, while the slope of *E*_es_ was steepened in denervated controls in response to bilateral carotid occlusion, this response was markedly attenuated in HU mice. In terms of vascular properties, actual and simulated microgravity induce changes in vessel transmural pressure due to the head distribution of blood, which could lead to structural and functional remodeling of systemic arteries. Vaziri et al. (2000) reported that HU rats exhibited an upregulation of inducible nitric oxide synthase protein expression in the thoracic aorta, and showed a depressed pressor response to norepinephrine compared with control rats. Furthermore, it is well known that actual and simulated microgravity could lead to 15%–18% plasma volume loss (Eckberg & Fritsch, 1991). In addition to these changes in heart and vascular properties, reduced stressed volume may also contribute to the significant decrease in the neuromechanical arc gain in HU rats.

### 4.3 Baroreflex equilibrium diagram in HU rats

**FIGURE 9:**
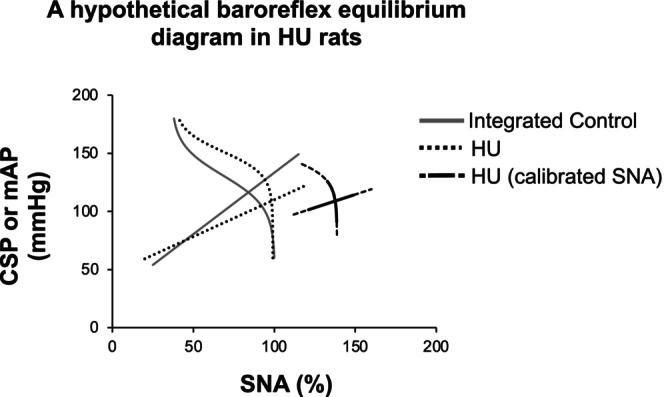
Illustration of a hypothetical baroreflex equilibrium diagram taking into account sympathoexcitation in hindlimb unloading (HU) rats. A rightward shift in the mechanoneural arc together with a downward shift in the neuromechanical arc are estimated to occur in HU rats (from black dotted lines to black solid lines), while there is no shift in integrated control rats (gray lines). CSP, carotid sinus pressure; mAP, mean arterial pressure; SNA, sympathetic nerve activity.

To understand the mechanisms that determine the SNA and AP responses in HU rats, we illustrated the baroreflex equilibrium diagram by plotting the mechanoneural and neuromechanical arcs on the same plane (Figure 7). As mentioned above, the mechanoneural arc was shifted toward a higher CSP range and the neuromechanical arc was shifted downwards in HU rats compared with integrated control rats. In addition to the significant decrease in the neuromechanical arc gain, the mechanoneural arc gain at the operating point also showed a significant decrease in HU rats (HU vs. integrated control: 0.28 ± 0.36 vs. 0.93 ± 0.57%/mmHg, *p* < 0.05). These alterations of the mechanoneural and neuromechanical arcs resulted in a significant decrease in the total loop gain of the baroreflex at the operating point [calculated from the product of the tangential slope of the mechanoneural arc and the slope of the neuromechanical arc (Table 3)]. However, the results shown in Figure 7 need to be interpreted with caution. Because the standardization of SNA was conducted in individual rats, a direct comparison of the absolute values of SNA between the two groups is not possible. In this study, the 24‐h urinary norepinephrine excretion was approximately 1.4 times higher in HU rats on Day 14 of HU compared with control rats. Taking into account the sympathoexcitation state in HU rats compared with control rats, we illustrated a hypothetical baroreflex equilibrium diagram for HU rats, showing a rightward shift of the mechanoneural arc and a downward shift of the neuromechanical arc, as shown in Figure 9. Because there is no evidence indicating a strict linear relationship between urinary norepinephrine excretion and the absolute values of SNA, we were not able to calibrate SNA quantitatively. Therefore, we drew the hypothetical baroreflex equilibrium diagram only around the operating point.

### 4.4 Physiological implications

Open‐loop analysis of the baroreflex showed a significant reduction of the total loop gain of baroreflex at the operating point in HU rats compared with integrated control rats. We previously reported that the baroreflex attenuates the pressure disturbance at 0.01–0.1 Hz; the baroreflex functioning frequency range in the power spectrum analysis (Mannoji et al., 2019). Additionally, sinoaortic denervation presented increased AP variability and widened the histograms of AP. Therefore, our result could explain the AP lability revealed by SD and histogram analyses of HU rats after hindlimb unloading and reloading in protocol 1. On the other hand, the locomotor activity of the rats can affect the width of the histogram. Tsvirkun et al. (2012) reported that HU rats showed hypokinesia for only the first few days during the nighttime, while a further increase in the number of movements during the second week of unloading was observed. Thus, the locomotor activity after hindlimb unloading might partly affect the changes in the histograms of AP.

Furthermore, our numerical simulation study indicates that hypotensive stress (−30 mmHg) induces a significant reduction in the total loop gain of baroreflex, resulting in a significantly diminished AP response in HU rats compared with integrated control rats. While the total loop gain at the operating point decreased by about 60%, from 1.1 ± 0.5 mmHg/mmHg to 0.5 ± 0.4 mmHg/mmHg, even in integrated control rats by the hypotensive stress (−30 mmHg), that decreased by about 90%, from 0.3 ± 0.4 mmHg/mmHg to 0.03 ± 0.04 mmHg/mmHg, in HU rats. As a result, HU rats could not almost buffer the decrease of AP. The changes in hemodynamics induced by microgravity or simulated microgravity have been reported under baroreflex closed‐loop conditions. In the present study, we conducted the open‐loop analysis of the arterial baroreflex and elucidated the abnormal intersection of the mechanoneural and neuromechanical arcs in HU rats. Therefore, the attenuated pressure response against hypotensive stress is one of the candidate mechanisms causing post‐spaceflight OH. To prevent post‐flight OH, fluid and volume loading prior to landing is effective (Bungo et al., 1985). To counteract hypotensive stress, hypertensive stresses such as increasing circulating volume by infusion could shift the neuromechanical arc upward in the baroreflex equilibrium diagram. In this case, the operating point is shifted toward an increase in the gain of the mechanoneural arc. As a result, the total loop gain of baroreflex may increase, leading to orthostatic tolerance. Orthostatic hypotension is also often observed after prolonged bed rest in clinical settings (Tzur et al., 2019). Indeed, hindlimb unloading is a model used to mimic prolonged bed rest as well as microgravity. Therefore, the results of the present study may also be applied to clinical settings of OH caused by prolonged bed rest.

### 4.5 Limitations

There are several limitations in this study. First, HU reflects only simulated microgravity, while astronauts experience both microgravity during spaceflight and hypergravity 3–5 times the gravity on Earth in the landing phase (Jordan et al., 2022). Yanagida et al. (2014) reported that even mild hypergravity (1.5 G) reduced spontaneous cardiac BRS assessed by sequence slope and transfer function gain. Therefore, hypergravity also may affect the baroreflex‐mediated sympathetic AP regulation after reloading. Second, we cut the vagal nerves to allow open‐loop analysis of baroreflex. Considering the increase of post‐flight HR compared with pre‐flight, parasympathetic activity also may be affected by microgravity. Therefore, the influence of altered parasympathetic activity cannot be ignored when considering the mechanisms of post‐flight OH. Third, the hemodynamic responses against microgravity may be affected by the duration of microgravity exposure. Cardiac output and stroke volume increased by 35%–41% after 3–6 months of microgravity exposure, which were greater than during shorter spaceflights (Norsk et al., 2015). Although the post‐flight BRS was reported to be reduced irrespective of the duration of microgravity exposure (Cooke et al., 2000; Fritsch et al., 1992), the alterations of mechanoneural and neuromechanical arcs of baroreflex may be different between shorter and longer durations of microgravity exposure. As we studied the baroreflex function after only 2 weeks of HU, further investigations will be needed. Additionally, the baroreflex function may be different between males and females. Foley et al. (2005) reported that HU‐induced attenuated SNA response to hypotensive stimulus was further weakened in female rats compared with male rats. We used male rats in the present study: open‐loop analysis of baroreflex in female HU rats may show a greater decrease in total loop gain of baroreflex at the operating point. Furthermore, there were some differences in the way to house control rats and HU rats. While HU rats were housed individually in each cage, control rats without harness were housed in groups. Tsvirkun et al. (2012) reported that social isolation and restraint as well as hindlimb unloading caused significant perturbations in blood pressure and HR. Therefore, our present results may also partly reflect the effects of isolation and restraint in HU rats.

## 5 CONCLUSIONS

The total loop gain of baroreflex at the operating point decreased significantly in HU rats compared with control rats. Hypotensive stress further attenuated the total loop gain of baroreflex in HU rats. This may contribute partly to the mechanism of OH after microgravity exposure.

## AUTHOR CONTRIBUTIONS

K.K., H.M., and T.T. performed experiments; K.K., T.N., and K. Saku analyzed data; K.K., K. Sunagawa, and K. Saku interpreted results of experiments; K.K. prepared figures; K.K. and K. Saku drafted the manuscript; K.K., H.T., K. Sunagawa, and K. Saku edited and revised the manuscript; K.K., H.M., T.T., T.N., H.T., K. Sunagawa, and K. Saku approved the final version of the manuscript.

## FUNDING INFORMATION

This study was partly supported by grants from Grant‐in‐Aid for Scientific Research (JSPS KAKENHI 22K08222), the research program of Japan Agency for Medical Research and Development (24hk0102085h0003), the research program of the Ministry of Internal Affairs and Communications (SCOPE: JP225006004), the Intramural Research Fund for Cardiovascular Diseases of National Cerebral and Cardiovascular Centre (24‐B‐7), the research grant from JST (JPMJPF2018), and the research grant from NTT‐Research, Inc. The authors confirm that these parties did not influence the study design, contents of the article, or selection of this journal.

## CONFLICT OF INTEREST STATEMENT

Saku K received research funding from Abiomed Inc., NTT Research, Inc., Asahi Kasei ZOLL Medical Corporation, Neuroceuticals Inc., and Zeon Medical Inc., and honoraria from Abiomed Japan K.K. and Mallinckrodt Pharma K.K.

## Data Availability

The data can be provided upon reasonable request to the corresponding author.
